# Subcellular Dynamic Immunopatterning of Cytosolic Protein Complexes on Microstructured Polymer Substrates

**DOI:** 10.1021/acssensors.1c01574

**Published:** 2021-10-15

**Authors:** Roland Hager, Ulrike Müller, Nicole Ollinger, Julian Weghuber, Peter Lanzerstorfer

**Affiliations:** † University of Applied Sciences Upper Austria, School of Engineering, 4600 Wels, Austria; ‡ Austrian Competence Centre for Feed and Food Quality, Safety & Innovation, Head Office: FFoQSI GmbH, Technopark 1C, 3430 Tulln, Austria

**Keywords:** micropatterning, cytosolic protein complexes, COP substrate, dynamic immunopatterning, EGFR

## Abstract

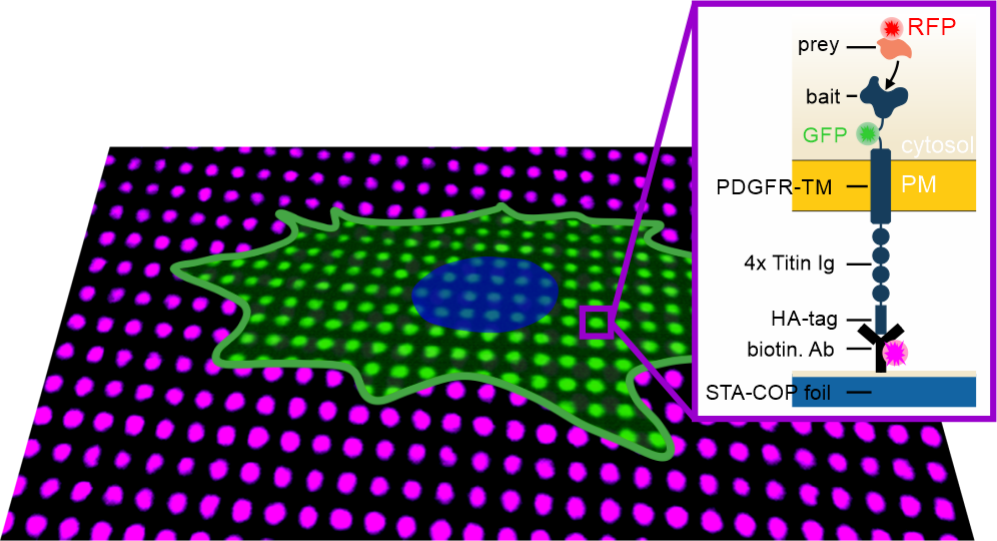

Analysis of protein–protein interactions in living cells by protein micropatterning is currently limited to the spatial arrangement of transmembrane proteins and their corresponding downstream molecules. Here, we present a robust and straightforward method for dynamic immunopatterning of cytosolic protein complexes by use of an artificial transmembrane bait construct in combination with microstructured antibody arrays on cyclic olefin polymer substrates. As a proof, the method was used to characterize Grb2-mediated signaling pathways downstream of the epidermal growth factor receptor (EGFR). Ternary protein complexes (Shc1:Grb2:SOS1 and Grb2:Gab1:PI3K) were identified, and we found that EGFR downstream signaling is based on constitutively bound (Grb2:SOS1 and Grb2:Gab1) as well as on agonist-dependent protein associations with transient interaction properties (Grb2:Shc1 and Grb2:PI3K). Spatiotemporal analysis further revealed significant differences in stability and exchange kinetics of protein interactions. Furthermore, we could show that this approach is well suited to study the efficacy and specificity of SH2 and SH3 protein domain inhibitors in a live cell context. Altogether, this method represents a significant enhancement of quantitative subcellular micropatterning approaches as an alternative to standard biochemical analyses.

Protein micropatterning has become an important tool for fundamental research in cell biology. Micropatterned substrates were mainly engineered for the investigation of the influence of the extracellular environment on cell morphology, cell differentiation, cytoskeleton rearrangement, cell migration, and organelle positioning.^1,2^ Within this regard, soft lithography via microcontact printing (μCP) is one of the most convenient and widely used methods for patterning proteins on a micron- and even nanometer-scale.^3−5^

Recently, others and we have developed protein micro- and nanopatterning approaches on solid substrates for the quantitative investigation of protein–protein interactions (PPIs) in the live cell.^4,6−14^ The fundamental strategy of these approaches is the spatial rearrangement of a cell surface protein (“bait”, e.g., receptor) in the shape of the printed patterns within the plasma membrane (e.g., by use of antibodies or ligands) and the monitoring of the lateral distribution of a putative interaction partner (“prey”, e.g., cytosolic adapter protein). This enables the investigation of PPIs in a native environment and membrane composition, and importantly, allows for straight-forward characterization of PPIs in the living cell. However, these methods are mostly limited to protein complexes formed between solely membrane-anchored bait and prey molecules or with an interacting intracellular prey.

As cytosolic protein complexes play a key role in the precise regulation of cellular signaling events, they have become putative new selective drug targets.^15^ Hence, there is an increasing interest in the design and development of robust experimental approaches beyond standard biochemical methods (e.g., such as co-immunoprecipitation, pull-down experiments, and yeast two-hybrid screens) that enable in-depth characterization of protein complexes inside cells. Information on interaction properties such as binding affinities, lifetimes, stability, and dynamics of protein complex formation is of particular importance, as these parameters are critical for the regulation of cellular systems.^16^

Within this regard, an *in situ* single-cell pull-down approach on micropatterned functionalized surface architecture in combination with single-molecule fluorescence imaging was developed to measure protein complex stoichiometry and dynamics.^17^ Recently, a microfluidic device for *in situ* co-immunoprecipitation of target proteins to detect PPIs in individual cancer cells with high fidelity was reported.^18^ Furthermore, a single-molecule pull-down assay was described which enables direct visualization of individual cellular protein complexes by single-molecule fluorescence microscopy.^19^ However, those sophisticated approaches have in common that cells cultured on these supports need to be lysed by detergents prior to PPI analysis and therefore do not allow for live cell measurements. In order to analyze PPIs in the cytosol of living cells, Gandor et al.^20^ reported a strategy using artificial receptor constructs (termed bait-PARCs) that transfer a micrometer-scale antibody surface pattern into an ordered array of cytosolic bait proteins in the plasma membrane. Most recently, a similar approach for real-time quantification of cytosolic PPIs using cell-based molography as a biosensor was developed.^21^ In addition, subcellular micropatterning of artificial transmembrane receptors was proved by using fibrinogen anchors.^22^

Based on the most recent developments, which also demonstrate the importance and future applicability of micropatterned interfaces for intracellular PPI analysis, we here report a robust platform for dynamic immunopatterning of cytosolic PPIs. The approach is based on subcellular micropatterning of bait-presenting artificial transmembrane constructs in the cytoplasm of living cells. We introduced a cyclic olefin polymer (COP) as a cost-saving and flexible alternative to glass coverslips for large-area μCP and realized a 384-well plate-based platform with modular protein micropatterns which enabled an increased experimental throughput. Furthermore, we redesigned and optimized bait-presenting artificial receptors (herein referred to as bait-PARs) for enhanced prey corecruitment. In order to demonstrate the applicability of the method, we investigated cytosolic protein complexes downstream of the epidermal growth factor receptor (EGFR). The EGFR has become one of the most extensively studied cell surface receptors and a major oncogenic drug target, as aberrant receptor activation and intracellular signal transduction is associated with a variety of cancers, thus making its key players in downstream signaling to the perfect proof of concept target for our study.^23^ We could unequivocally show that EGFR downstream signaling is based on Grb2-mediated ternary protein complexes exhibiting different interaction regimes (constitutively bound *vs* agonist-dependent). Additionally, we identified significant differences in protein complex formation kinetics and stability of detected assemblies, which might account for the dynamic regulation of normal and aberrant EGFR signaling. Furthermore, we characterized the efficacy and specificity of therapeutic Src homology (SH) domain inhibitors with high sensitivity.

Altogether, we could demonstrate that our technology allows for the control of the subcellular localization of cytosolic adapter proteins, hence enabling the spatiotemporal investigation of receptor-mediated intracellular PPIs within a defined signaling cascade. With the introduction of this robust and flexible assay, we introduce an add-on to standard biochemical PPI analysis, which might facilitate protein micropatterning for cell biological investigations in the future.

## Results and Discussion

### Fabrication of Micropatterned COP Foils Using Large-Area μCP

We have recently introduced large-area patterned glass substrates with modular protein micropatterns that enable the systematic investigation of specific and nonspecific effects in the analysis of PPIs in adherent cells.^9^ However, functionalized glass substrates possess major drawbacks such as increased specific costs and high fragility, especially when used in combination with sensitive fluorescence spectroscopy approaches such as total internal reflection fluorescence (TIRF) microscopy, as they require a glass thickness below 200 μm (“coverslip”). As a cost-saving and flexible alternative, we recently described COP foils for the fabrication of micropatterned substrates based on a photolithographic approach.^24^

**Figure 1:**
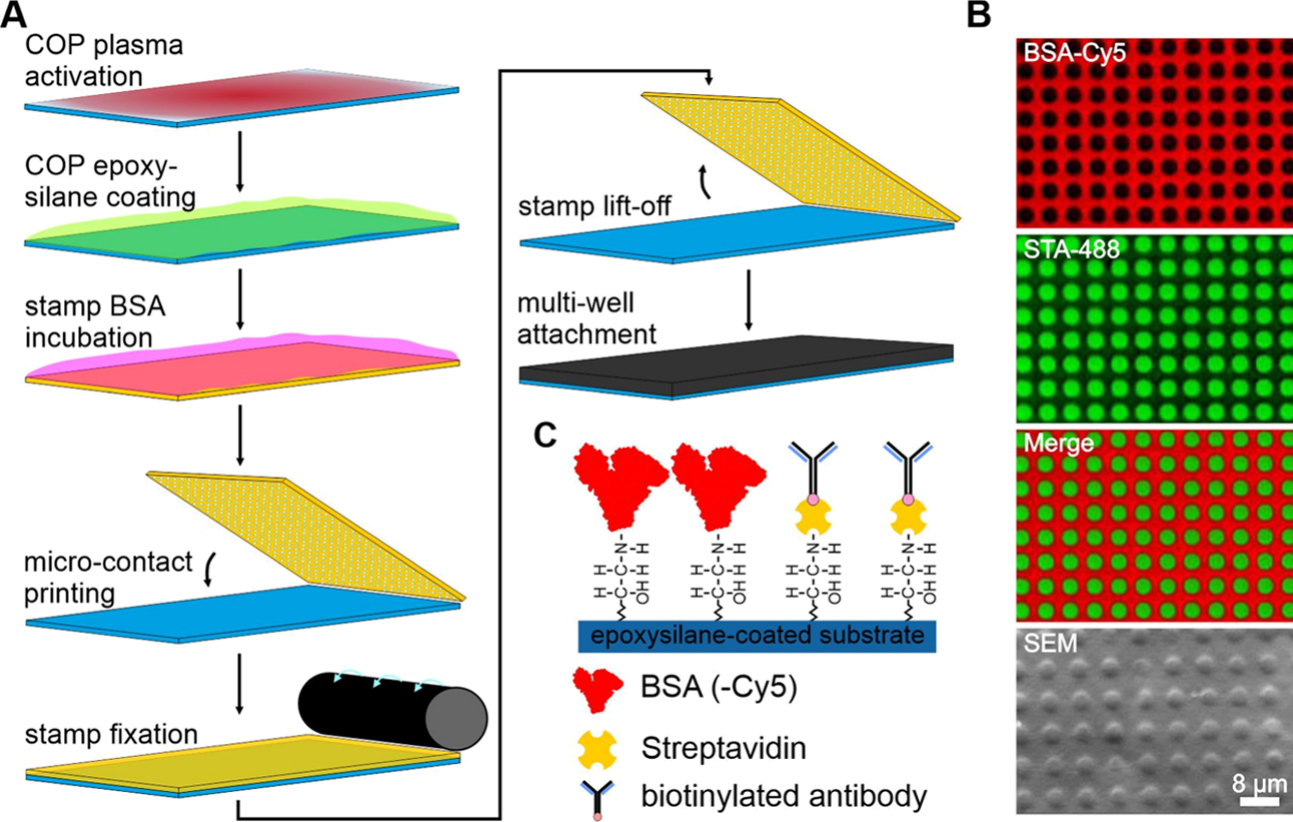
Overview of μCP on COP substrates. (A) Schematic workflow of the μCP procedure. In short, COP foils are activated by air-plasma oxidation followed by the introduction of epoxide functional groups. Next, a large-area PDMS stamp containing a continuous grid pattern with a feature size and depth of 3 μm is incubated with a BSA-Cy5 (or BSA) solution for surface passivation. After a washing step, the stamp is placed onto the substrate by its own weight. After stripping off the stamp, the functionalized COP substrate is bonded with a 384-well plastic casting. (B) Detailed section of the ready-to-use 384-well plate surface for live-cell experiments. Representative TIRF microscopy images of the BSA-Cy5 grid (red, top), Alexa Fluor 488 conjugated streptavidin (green, middle), and merged images (bottom) are shown. (C) Schematic drawing of surface functionalization for live cell experiments consisting of passivated BSA grid, covalent streptavidin-binding in between and addition of biotinylated anti-bait antibodies for specific bait-capturing.

Here, we present a technological extension of this method for functionalization of COP substrates using large-area polydimethylsiloxane (PDMS)-based elastomeric stamps. The fabrication process of the micropatterned COP foils using large-area μCP is depicted in Figure 1. To generate a substrate surface with a high density of oxygen-containing functional groups, such as carboxyl and hydroxyl groups, the COP foil was air-plasma activated in an initial step. Next, a multipurpose layer of epoxide functional groups was created for subsequent covalent biomolecule binding. The protein-patterned cell substrate was finally produced by printing a micrometer-sized BSA grid (for surface passivation) on the epoxysilane-coated COP surface. In order to compensate for the low rigidity of the COP foil, the patterned substrate was bonded with a 384-well plastic casting (Figure 1A), resulting in a ready-to-use multi-well plate that can be further functionalized in a modular manner for subsequent analysis of PPIs in live cells with high experimental throughput. Figure 1B shows TIRF microscopy images as well as a scanning electron microscope recording of a representative 3 μm BSA-Cy5 micropatterned COP surface. The fill-up with Alexa Fluor 488 conjugated streptavidin demonstrates the highly specific binding of proteins in the non-passivated regions. A schematic illustration of the surface chemistry and functionalization with streptavidin and biotinylated antibodies as a basis for subsequent cell experiments is depicted in Figure 1C.

To the best of our knowledge, we demonstrate the first approach for PDMS-based large-area μCP of biomolecules on a COP substrate with a micrometer resolution. We are certainly aware that μCP has several methodological limitations, mainly with respect to selectivity (how much protein is adsorbed by the substrate), homogeneity (how much the protein density within the patterned regions varies), and flexibility (each PDMS layout stamp needs a separate mask; and μCP of multiple proteins is difficult due to alignment problems). However, μCP also provides some unrivalled properties compared to other, more sophisticated protein patterning technologies, especially in combination with our high-content micropatterning platform. By use of a customized silicon master (100 mm in diameter) containing a full array of round-shaped pillars with a feature size and a depth of 3 μm, we were able to create a large microstructured PDMS stamp for subsequent straightforward functionalization of COP foils. PDMS itself has various advantageous properties for a stamp material: (i) it possesses a hydrophobic surface with low surface energy (favorable for protein transfer onto the target surface), (ii) it is chemically inert and elastomeric (molds with high fidelity and can be easily removed from the mask as well as from the substrate), (iii) it can be reused, (iv) it is cheap, and (v) stamping with PDMS is comparatively easy to perform.^4,25,26^ Most importantly, our protein patterning approach is robust, highly reproducible, easy to implement (no special and expensive laboratory equipment is necessary), and tremendously fast (30 min PDMS inking with protein solution and stamping overnight followed by a bonding step). Additionally, functionalized substrates can be further modified in a modular manner (e.g., with DNA-based systems).^9^

### Experimental Strategy and Optimization for Profiling Cytosolic Protein Complexes in the Live Cell

**Figure 2:**
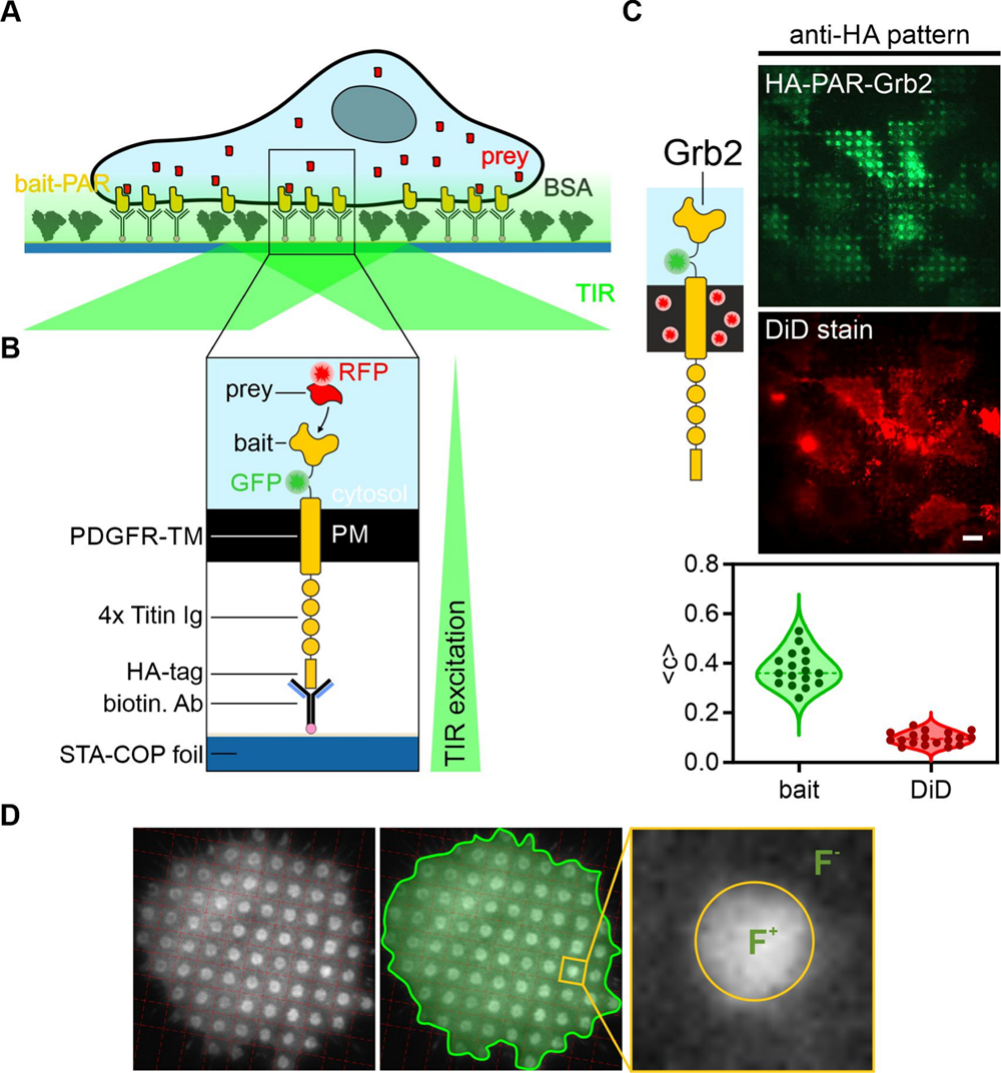
Bait-presenting artificial receptors (bait-PARs) for dynamic immunopatterning of cytosolic protein complexes. (A) Schematic presentation of the micropatterning assay. Cells are transiently co-transfected with bait-PARs fused to GFP (or RFP) and RFP-labeled (or GFP) prey molecules. Upon specific antibody–antigen interactions, bait-PARs are rearranged in the plasma membrane according to the micrometer-scale antibody pattern on the COP substrate. The interaction between bait-PARs and the prey is monitored by the degree of prey copatterning. (B) Schematic illustration of a single bait-PAR. The bait-PAR is composed of an intracellular bait protein, a conjugated fluorophore, a single transmembrane domain, an extracellular spacer domain (four repeats of the Titin Ig domain I27), and a HA epitope tag, which directs the bait-PAR toward the pattern of the cognate immobilized anti-HA antibody. (C) Adaption of the bait-PAR assay for analysis of cytosolic protein complexes downstream of the EGFR. The bait protein (regulatory subunit of protein kinase A) of the previously published bait-PAR^20^ was exchanged with the growth-factor receptor binding protein 2 (Grb2). In order to proof sufficient cell attachment to micropatterned COP substrates as a prerequisite for TIRF microscopy, HeLa cells were transiently transfected with HA-PAR-Grb2 and cell membrane was stained with the lipophilic tracer DiD. Scale bar: 9 μm. Violin plot depicts min to max values of fluorescence bait-PAR-Grb2 or DiD contrast of 16 analyzed cells. (D) Procedure of contrast calculation. An automated gridding algorithm detects pattern elements inside the cells and calculates the grid-size and rotation angle (left). Cells to be analyzed are then selected manually (middle) and fluorescence contrast can be calculated for each pattern (right) based on the ratio of the average intensity of the inner pixels of the pattern and the pixels surrounding the pattern. Abbreviations: biotin. Ab, biotinylated antibody; HA-tag, human influenza HA epitope tag; PDGFR-TM, transmembrane domain of PDGF receptor; PM, plasma membrane; STA-COP foil, streptavidin-coated COP foil.

Based on this antibody patterning approach, we have recently investigated PPIs between various membrane-anchored bait and intracellular prey molecules.^8−10,27,28^ To expand this method for the analysis of exclusively cytosolic PPIs, we adopted the approach of Gandor et al.^20^ and further developed it for investigation of a broadened spectrum of intracellular PPIs with enhanced experimental throughput (Figure 2). We therefore combined the use of bait-presenting artificial receptors with our modular and robust large-scale protein patterning platform (Figure 2A). The bait-PAR consists of (i) a selected intracellular bait protein, (ii) an inert transmembrane domain (PDGF receptor transmembrane domain), and (iii) a flexible extracellular domain (four repeats of Titin Ig I27 domain) that contains a human influenza hemagglutinin (HA) epitope tag, which directs the artificial receptor toward the patterned anti-HA antibodies (Figure 2B). The bait-PAR as well as the cytosolic prey are expressed as a fluorescent fusion protein and PPIs are monitored by the degree of bait–prey copatterning using TIRF microscopy. In order to exemplify the validation and broad applicability of this assay, we constructed a bait-PAR consisting of the growth factor receptor-bound protein 2 (Grb2), herein referred to as bait-PAR-Grb2. Grb2 is a widely expressed cytosolic adapter protein and acts as an intermediate between cell–surface activated receptors and downstream targets through SH2 and SH3 domains.^29^ Furthermore, Grb2 is reported to mediate intracellular signaling dynamics by the interaction with a variety of downstream molecules,^30^ and therefore represents a perfect intracellular proof of concept target. In a first attempt, the correct bait-PAR-Grb2 orientation across the cell membrane as well as the cell membrane–substrate interface was investigated as prerequisites for further analysis using TIRF microscopy. For this purpose, GFP-fused bait-PAR-Grb2 was transiently expressed in HeLa cells, which were subsequently incubated on an anti-HA antibody patterned COP substrate (Figure 2C). For quantitation of the lateral bait and prey distribution, the respective fluorescence signal intensities within and outside the antibody-patterned areas were compared (Figure 2D). The fluorescence contrast (signal ratio) is averaged over all patterns within single cells and provides a measure for the specificity of bait enrichment as well as for the bait–prey interaction strength.^13^ Bait-PAR-Grb2 was found to be significantly enriched in the cognate antibody-functionalized micropatterns (mean fluorescence contrast ⟨*c*⟩ 0.38 ± 0.02), indicating a correct integration of the artificial construct into the plasma membrane as well as a high specificity of antigen–antibody binding. On the contrary, we observed a homogenous staining with the lipophilic dye DiD (mean fluorescence contrast ⟨*c*⟩ 0.09 ± 0.01), demonstrating a flat interface of the cell membrane with the patterned COP substrate to avoid false positive results. As HeLa cells were shown to fulfil those requirements, they were used throughout the study. Compared to the control conditions, we detected a ∼4-fold increase in bait protein enrichment in antibody patterned areas. This value is comparable to other studies using different protein patterning approaches,^6,22,31^ again proving the effective surface functionalization of our platform.

**Figure 3:**
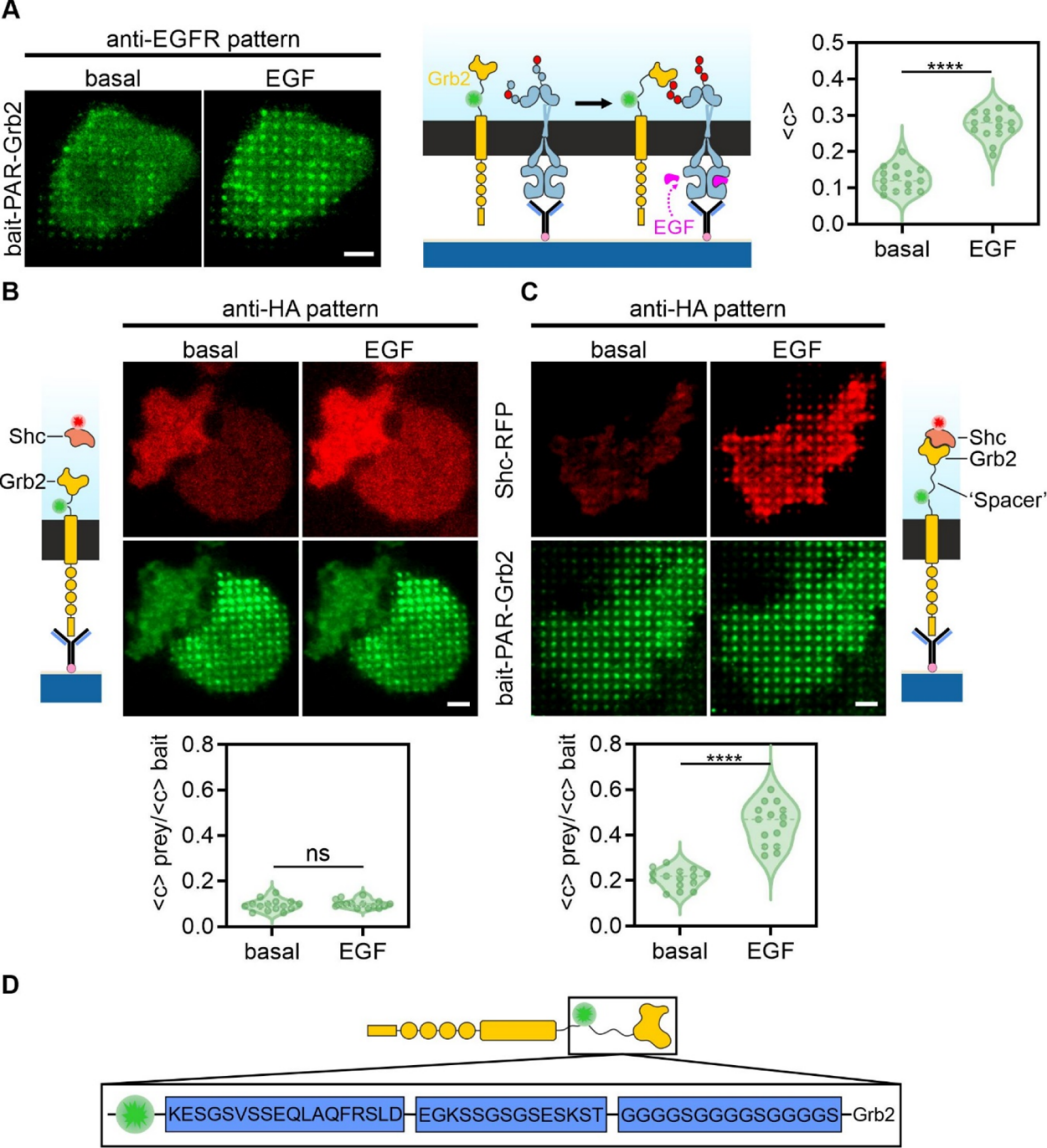
Test of bait functionality coupled to artificial receptor construct and optimization for protein complex formation. (A) Cells expressing modified bait-PAR consisting of wildtype Grb2 were grown on anti-EGFR patterned surfaces. Copatterning of bait-PAR-Grb2 to EGFR-enriched micropatterns was analyzed and quantitated before and after EGF stimulation (170 nM, 10 min). Scale bar: 15 μm. (B) Cells co-expressing bait-PAR-Grb2 (GFP-fused, green) and Shc-RFP (RFP-fused, red) were grown on anti-HA patterns and copatterning of Shc to bait-PARs upon EGF stimulation (170 nM, 10 min) was assessed by TIRF microscopy. Representative images of cells expressing adapted bait-PAR-Grb2 (B) and optimized bait-PAR-Grb2 with additional amino acid sequences to enhance flexibility of the bait protein (C). Scale bar: 12 μm. (D) Schematic drawing of optimized bait-PAR-Grb2 with inserted linker sequences between fluorophore and bait protein. Violin plots depict min to max values of fluorescence contrast (A) or bait-normalized prey contrast (B,C) of 15 analyzed cells. ****p < 0.0001 for comparison of prey copatterning before and after EGF stimulation. ns, no significant differences.

In a next step, the functionality of Grb2 fused to the bait-PAR was tested (Figure 3). Therefore, we used the well-described interaction between the EGFR and Grb2. As Grb2 has been reported to directly bind phosphotyrosine (pTyr)-containing sequences on the EGFR via its SH2 domain,^32,33^ we used the EGFR as the bait and Grb2 coupled to the artificial receptor construct as the prey (Figure 3A). Cells expressing GFP-labeled bait-PAR-Grb2 were grown on an anti-EGFR patterned surface and were imaged using TIRF microscopy before and after EGF stimulation. The degree of bait-PAR-Grb2 copatterning to EGFR-enriched areas served as a parameter of EGFR downstream signaling activation. Under basal conditions, bait-PAR-Grb2 showed minor colocalization (⟨*c*⟩ 0.12 ± 0.01) with EGFR-enriched areas, whereas a significant copatterning was detected upon EGF stimulation within minutes (⟨*c*⟩ 0.27 ± 0.01, *p* < 0.0001), indicating that Grb2, despite coupled to the artificial transmembrane domain, can still translocate and bind to the ligand-activated EGFR.

To proof Grb2 activation and mediation of downstream signaling, we next investigated the ability of bait-PAR-Grb2 to corecruit further adapter proteins such as the SHC-transforming protein 1 (Shc1), which has been reported to be recognized by the Grb2 SH2 domain,^34^ similar to the EGFR. Therefore, cells coexpressing bait-PAR-Grb2 (GFP-fused) and Shc1-RFP were grown on anti-HA patterned surfaces and Shc1 copatterning was monitored upon EGF stimulation (Figure 3B). Surprisingly, we could not detect any significant Shc1 colocalization to the bait-PAR-Grb2 patterned areas, neither in unstimulated (⟨*c*_prey/bait_⟩ 0.09 ± 0.02) nor in EGF stimulated (⟨*c*_prey/bait_⟩ 0.11 ± 0.03) cells. However, we found a substantial RFP fluorescence intensity increase under TIR illumination conditions upon EGF addition, indicating an agonist-dependent Shc1 translocation to the cell membrane. To further elaborate on this issue, we intended to enhance the flexibility of the intracellular portion of the bait-PAR by inserting flexible fusion protein linkers between the fluorophore and Grb2 (Figure 3D).^35^ Indeed, in cells coexpressing the optimized bait-PAR-Grb2 and Shc1-RFP, a prominent Shc1 copatterning was detected upon EGFR activation (⟨*c*_prey/bait_⟩ 0.21 ± 0.01 *vs* ⟨*c*_prey/bait_⟩ 0.45 ± 0.02, *p* < 0.0001), indicating Grb2:Shc1 protein complex formation (Figure 3C). We therefore used the optimized and more flexible bait-PAR-Grb2 for subsequent experiments. A similar linker system was recently reported, investigating the Grb2:SOS1 complex by focal molography.^21^

Protein micropatterning can lead to the formation of protein clusters within or at the cell membrane with subsequent recruitment of relevant proteins,^22^ including bait and prey molecules of interest.^9^ To investigate unspecific bait-prey copatterning in the presented approach, bait-PAR and prey distribution was checked on microstructured surfaces but without antibody incubation (Figure S1). Neither under basal conditions nor after EGF stimulation, an unspecific copatterning was detected for all bait and prey proteins under the study. We therefore conclude that bait–prey copatterning occurs due to interactions and active corecruitment.

### Dynamic Immunopatterning Reveals Differences in Grb2-Mediated Protein Assemblies Downstream of the EGFR

**Figure 4:**
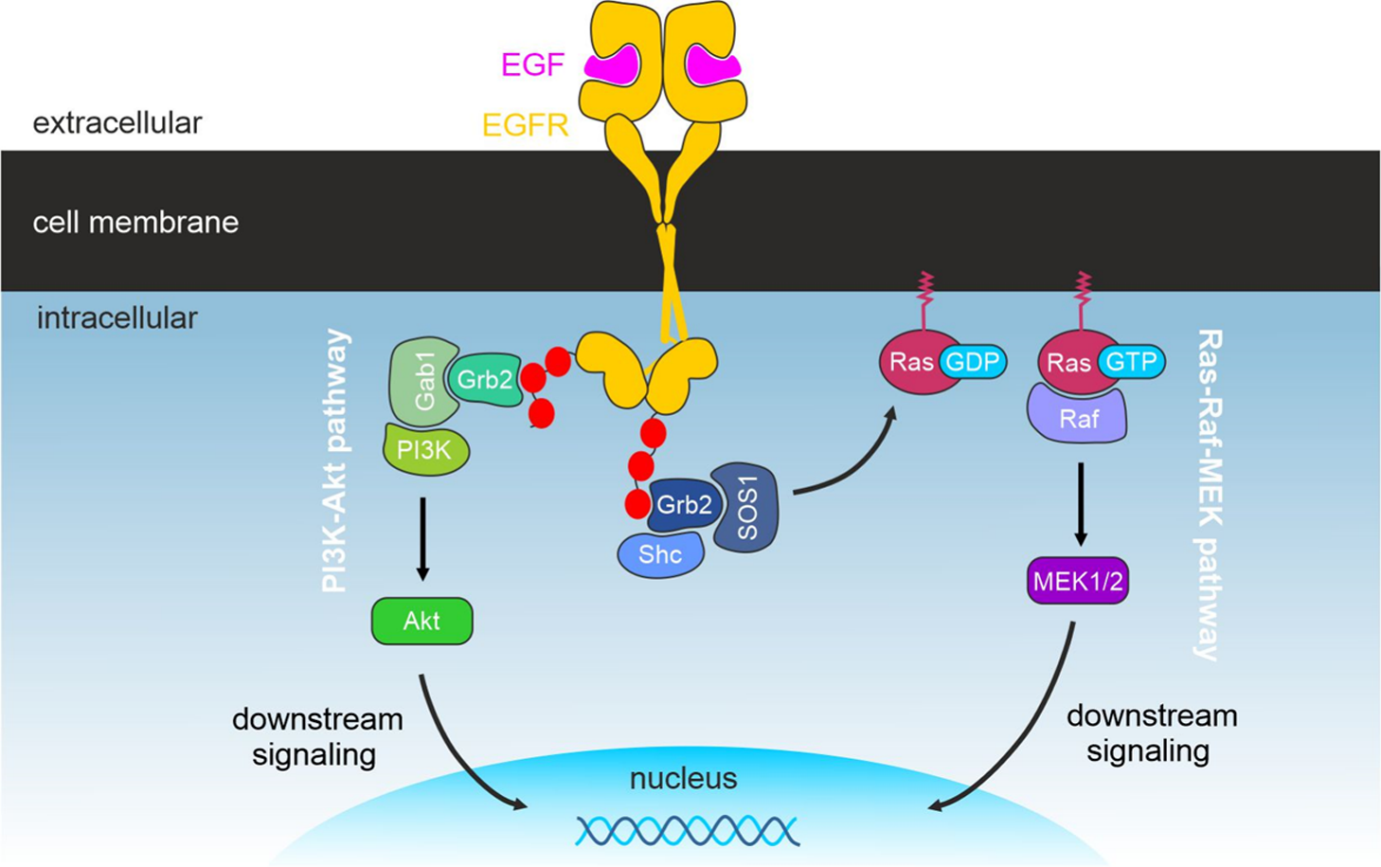
Illustration of the two main pro-oncogenic EGFR downstream signaling cascades and ligand-induced assembly of protein complexes. The Ras-Raf-MEK and the PI3K-Akt pathways are depicted. Adapted from Wee et al., 2017, Cancers.

**Figure 5:**
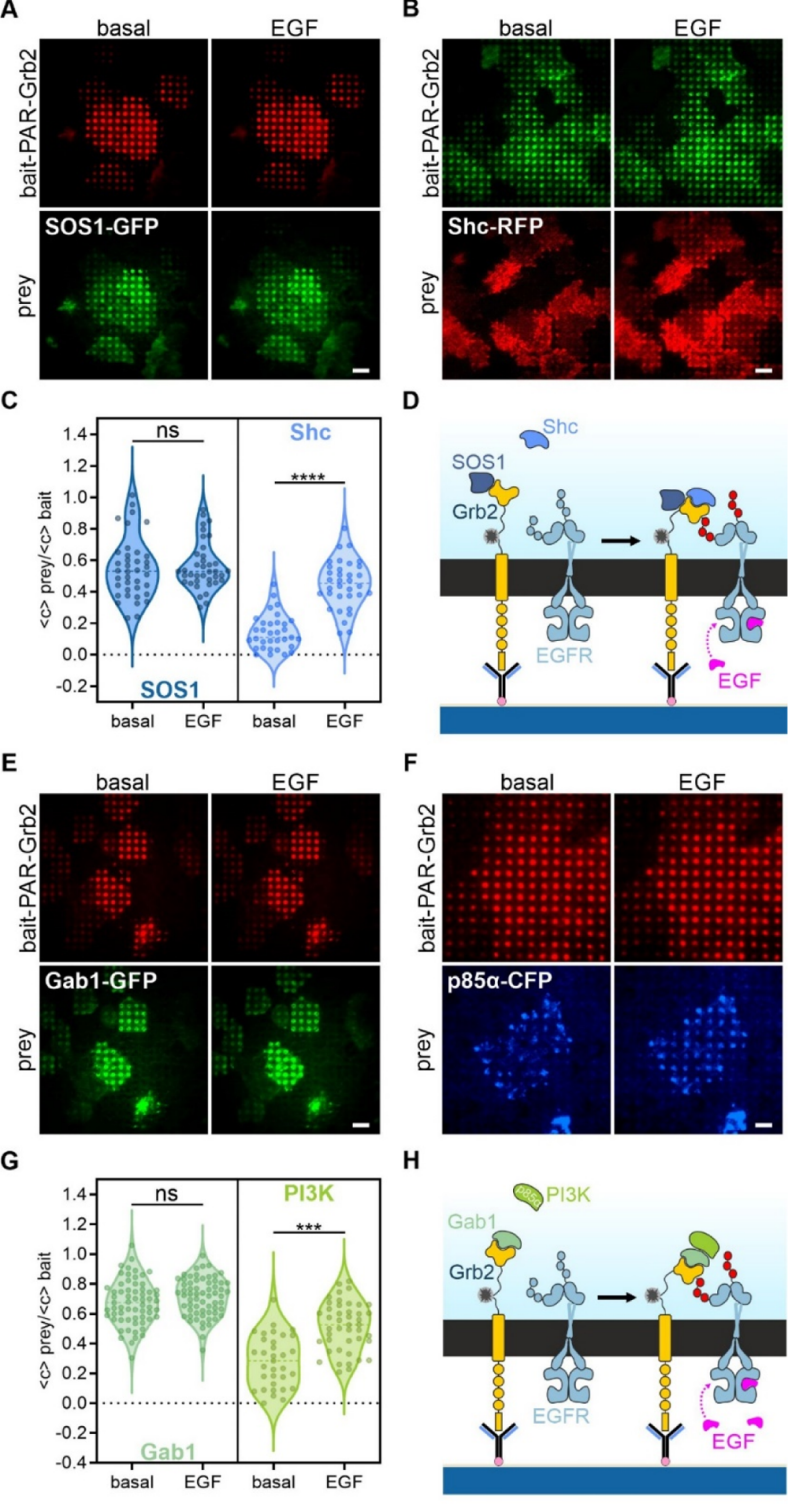
Dynamic immunopatterning reveals differences in EGFR-mediated cytosolic protein complexes. Initial signaling complexes of Ras-Raf-MEK (A–D) and PI3K-Akt pathway (E–H). HeLa cells were transiently co-transfected as the following: (A) bait-PAR-Grb2-RFP + SOS1-GFP, (B) bait-PAR-Grb2-GFP + Shc-RFP, (E) bait-PAR-Grb2-RFP + Gab1-GFP, and (F) bait-PAR-Grb2-RFP + p85α-CFP. Transfected cells were grown for at least four hours on anti-HA antibody patterned substrates 24 h after transfection. Representative TIRF microscopy images of cells expressing fluorescently labeled bait and prey proteins before and after EGF stimulation for 10 min (170 nM) (A,B,E,F) are shown. Scale bar: 9 μm. Schematic presentations illustrate indicated protein complex assembly (D,H). Violin plots show quantitation of bait-normalized fluorescence contrast of respective prey copatterning before and after EGF addition of at least 35 analyzed cells measured on three different days (C,G). ***p < 0.001 and ****p < 0.0001 for comparison of bait-normalized prey copatterning before and after EGF stimulation; ns, no significant differences.

The EGFR is a tyrosine kinase and is found to be upregulated in different types of cancers, mainly caused by mutations and truncations of its extracellular as well as its intracellular kinase domain. Consequently, the two main pro-oncogenic downstream signaling pathways, the Ras-Raf-MEK and PI3K-Akt pathway, are frequently over-activated.^36^ Hence, it is of critical importance to understand the molecular mechanisms that regulate EGFR signal transduction. Within this regard, cytosolic proteins downstream of the EGFR are attracting attention as key regulatory targets, particularly Grb2, as it is one of the most important proteins participating in EGFR signaling. Grb2 serves as an universal adapter protein once the EGFR is activated, subsequently leading to the activation of the aforementioned pro-oncogenic signaling pathways.^37^ To analyze Grb2-mediated protein complexes with high fidelity in a live cell context, we aimed for the dynamic immunopatterning of protein assemblies within the Ras-Raf-MEK and PI3K-Akt pathway by use of the bait-PAR system (Figures 4 and 5).

The Ras-Raf-MEK signal transduction pathway is initiated by EGFR activation through binding of its cognate ligands (EGF and transforming growth factor α), leading to EGFR dimerization and activation of its cytoplasmic tyrosine kinase domain.^23^ Subsequently, a ternary complex consisting of Shc1:Grb2:SOS1 (son of sevenless protein 1) is recruited to the phosphorylated RTK,^38^ which further leads to the activation of the membrane-bound small GTPase protein Ras (rat sarcoma protein). Upon exchanging GDP for GTP, Ras in turn activates the serine/threonine-specific protein kinase Raf (rapidly accelerated fibrosarcoma protein), leading to sequential phosphorylation and activation of the respective downstream signaling cascade.^39^

We first investigated the initial protein complex formation within the Ras-Raf-MEK pathway between Grb2, SOS1, and Shc1. Grb2 is known to be constitutively bound to SOS1, predominantly via its N-terminal SH3 domain,^40^ whereas Shc1 associates with Grb2 upon EGFR stimulation via the SH2 domain^41^ (Figure 5A–D). In cells coexpressing RFP-fused bait-PAR-Grb2 and SOS1-GFP, we indeed found a prominent SOS1 copatterning to bait-enriched micropatterns under basal conditions (⟨*c*_prey/bait_⟩ 0.55 ± 0.03), which did not change upon EGF stimulation (⟨*c*_prey/bait_⟩ 0.56 ± 0.02), again indicating an agonist-independent stable association between Grb2 and SOS1 (Figure 5A and C). On the contrary, in cells coexpressing GFP-fused bait-PAR-Grb2 and Shc1-RFP, we could confirm the agonist-dependent Grb2:Shc1 complex formation as indicated by a low degree of copatterning in unstimulated cells (⟨*c*_prey/bait_⟩ 0.14 ± 0.02), and a significant increase in Shc1 corecruitment (*p* < 0.0001) upon EGF stimulation (⟨*c*_prey/bait_⟩ 0.45 ± 0.03) (Figure 5B,C).

Besides the Ras-Raf-MEK signaling, the PI3K-Akt pathway is the second major EGFR-mediated signal transduction pathway, involving binding of Grb2 to the EGFR and subsequent association with Gab1 (Grb2-associated-binding protein 1; predominantly via the C-terminal SH3 domain) and the p85 subunit of PI3K (phosphoinositide 3-kinases; via pTyr residues of Gab1), resulting in the production of phosphatidylinositol (3,4,5)-triphosphate (PIP3) and activation of Akt.^36^ Like SOS1, Gab1 forms a constitutive complex with Grb2,^42^ whereas the association between Grb2:Gab1 and PI3K-p85 can be enhanced by EGF addition^43^ (Figure 5E–H). Again, we could detect a constitutive Grb2:Gab1 complex formation in cells coexpressing RFP-fused bait-PAR-Grb2 and Gab1-GFP, as indicated by the prominent Gab1 copatterning under basal conditions (⟨*c*_prey/bait_⟩ 0.67 ± 0.02) (Figure 5E,G). Similar to SOS1, the Gab1 fluorescence contrast did not change significantly upon EGFR activation (⟨*c*_prey/bait_⟩ 0.71 ± 0.02). For the investigation of PI3K association, we coexpressed the CFP-fused p85α regulatory subunit of PI3K and analyzed the copatterning to bait-PAR-Grb2 (Figure 5F,G). The Grb2:Gab1 complex readily showed an association with p85α in the absence of growth factor (⟨*c*_prey/bait_⟩ 0.28 ± 0.03), but this interaction was significantly enhanced by EGF addition (*p* < 0.001, ⟨*c*_prey/bait_⟩ 0.51 ± 0.02), suggesting further interaction between pTyr residues of Gab1 and p85α.

We next questioned whether those ternary protein complexes can actively recruit further downstream molecules. Therefore, the subcellular localization of proximate scaffold proteins such as Ras, Raf, and MEK1 (mitogen-activated protein kinase kinase 1) was investigated (Figure S2). Ras is activated by the guanine nucleotide exchange factor SOS1 by induction of the exchange of GDP to GTP.^36^ So far it is not clear whether this occurs through dissociation of the Grb2:SOS1 complex from the receptor and translocation to the membrane-bound Ras, or by active recruitment of Ras to the activated Grb2:SOS1 complex. As shown in Figure S2A, Ras can be actively copatterned to bait-PAR-Grb2 enriched areas, however, the majority of analyzed cells showed a homogenous HRas-CFP membrane distribution, indicating that Grb2:SOS1 or SOS1 alone dissociates from the receptor complex to activate Ras at the plasma membrane. Furthermore, a Grb2-independent SOS1 membrane-localization and receptor-triggered Ras activation has been recently reported,^44^ which could also explain our observation. Additionally, we cannot fully exclude a reduced SOS1:Ras interaction caused by spatial restrictions due to the artificially patterned Grb2:SOS1 complex. However, a similar appearance was also obtained for the downstream effector Raf, which is subsequently corecruited and activated by Ras.^36^ Raf1-CFP copatterning to bait-PAR-Grb2 patterns was a rather rare event, as in most of the cells Raf1 showed a homogenous membrane recruitment upon EGF stimulation, independently of bait-PAR-Grb2 micropatterns (Figure S2B). No copatterning was detected for MEK1-GFP, which in turn is activated by Raf (Figure S2C).

Intracellular signaling interactions are potentially much more complicated than the simplified models presented here. However, we could clearly show that our dynamic immunopatterning assay is suitable to generally characterize cytosolic protein complex formation. Moreover, we were able to confirm and to discriminate between constitutive protein complexes and agonist induced associations, which were mainly investigated by classical biochemical approaches such as co-immunoprecipitation in the past.

### Modulation of Cytosolic PPIs by Protein Complex Disruptors

**Figure 6:**
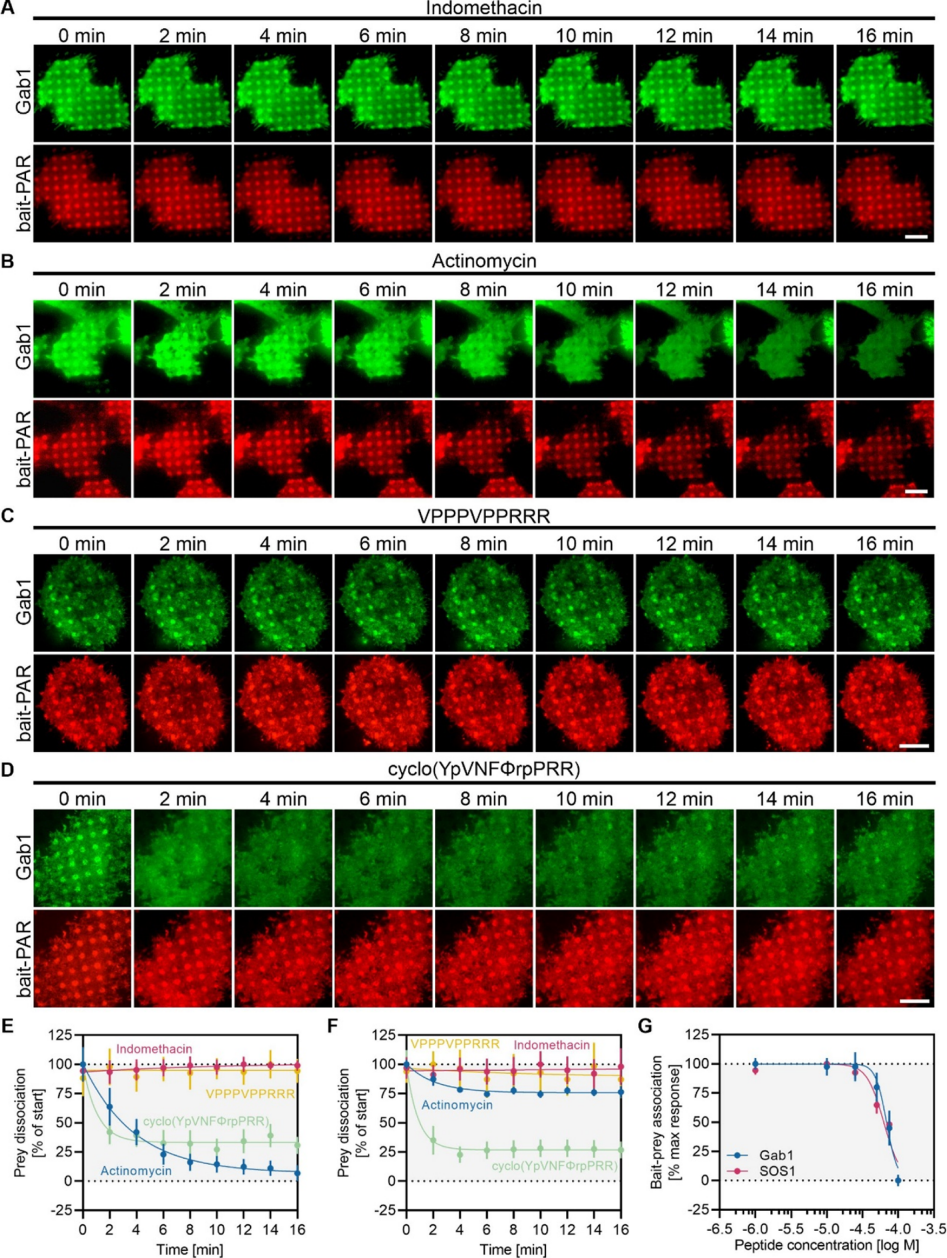
Disruption of protein complexes by protein domain inhibitors. Cells co-expressing bait-PAR-Grb2-RFP and Gab1-GFP were used to showcase the different effects of indicated inhibitory substances (A–D). Representative TIRF microscopy images show co-recruitment of the prey to bait micropatterns before and after pharmacological treatment. Scale bars: 15 μm. Quantitation of bait-normalized fluorescence contrast of Gab1 (E) and SOS1 (F) dissociation kinetics upon substance treatment. (G) Dose-response relationship of cell-permeable disruptive peptide. Data represent mean ± SE of >40 analyzed cells measured on at least two different days.

Recent studies evidenced that Grb2 is involved in the development and progression of multiple tumor malignancies such as breast, lung and bladder cancer, chronic myelogenous leukemia, hepatocellular carcinoma, and so forth.^45^ Therefore, Grb2 has become an attractive therapeutic target, mainly by modulating its downstream signaling activity by peptidomimetics via blocking its SH2 (connection to cell surface receptors via Shc1 interaction) and SH3 (interlink to downstream pathways) domains.^46−49^ To demonstrate the applicability of our assay to study PPI inhibitors in living cells, we monitored the dissociation behavior of the constitutively bound SOS1:Grb2:Gab1 ternary signaling complex (Figure 6).

To this end, HeLa cells expressing bait-PAR-Grb2-RFP and GFP-fused SOS1 or Gab1 were stimulated with various reported disruptive substances and bait–prey copatterning was monitored over time. To showcase the different effects of agents under study [10 μM indomethacin, 10 μM actinomycin D, 100 μM peptide VPPPVPPRRR and 100 μM peptide cyclo(YpVNFΦrpPRR)], representative TIRF microscopy images of cells co-expressing bait-PAR-Grb2-RFP and Gab1-GFP are depicted in Figure 6A–D. Indomethacin, a known nonsteroidal anti-inflammatory drug and recently identified inhibitor of the Shc1:EGFR interaction,^50^ was used as a negative control for the N- and C-terminal SH3 domain mediated SOS1:Grb2:Gab1 interaction. No effect on Gab1 (Figure 6A,E) and SOS1 (Figure 6F) copatterning was detected over a time period of 16 min. On the contrary, actinomycin D, a reported anti-cancer drug and Grb2 SH2 domain inhibitor,^51^ was identified also as a potent SH3 domain inhibitor, resulting in a gradual dissociation of Gab1 (Figure 6B,E) and SOS1 (Figure 6F) from Grb2. For Gab1, copatterning was remarkably reduced by ∼95% with a dissociation half-life of 2.6 min, whereas SOS1 copatterning was reduced by ∼25% (dissociation half-life of 1.4 min), indicating a more pronounced affinity of actinomycin D for the C-terminal SH3 domain of Grb2, which mediates binding of Gab1. In recent years, high affinity Grb2-binding peptides have been developed to block Grb2 association to cell surface receptors^52,53^ or binding to downstream molecules.^48,54^ We therefore further tested two known Grb2 inhibitors, the SH3 domain blocking peptide VPPPVPPRRR^55^ and the most recently described Grb2 SH2 domain inhibitor cyclo(YpVNFΦrpPRR).^56^ Upon stimulation with 100 μM VPPPVPPRRR, we could not detect any effect on Gab1 and SOS1 copatterning (Figure 6C,E,F). This observation might be readily explained by a general low lipid membrane permeability of peptides.^57^ Thus, extensive effort has been made to develop novel cyclic cell-penetrating peptides (CPPs) that are in addition capable of binding to target proteins with an antibody-like affinity and specificity, such as the CPP cyclo(YpVNFΦrpPRR).^56^ Indeed, when cells were treated with 100 μM cyclo(YpVNFΦrpPRR), we observed a rapid dissociation of Gab1 (Figure 6D,E) and SOS1 (Figure 6F) from Grb2, reaching a maximum prey dissociation of 70–75% already after 4–6 min of peptide treatment. The comparable dissociation half-life of 0.6 min (SOS1) and 0.7 min (Gab1) indicates a similar affinity of cyclo(YpVNFΦrpPRR) for the N- and C-terminal SH3 domains of Grb2. Our results indicate that the peptide cyclo(YpVNFΦrpPRR) does not only block the Grb2 SH2 domain as previously reported^56^ but also the SH3 domains, which indicates that the assay has the ability of identifying novel inhibitory targets. Peptide cyclo(YpVNFΦrpPRR) was reported to dose-dependently reduce the level of phosphorylated MEK (p-MEK) with an IC50 value of ∼15 μM. Therefore, we further elaborated on the half-maximal effective peptide concentration (EC50), which is necessary to dissolve the bait-prey interaction in our system (Figure 6G). In line with the comparable dissociation properties, we observed similar EC50 values for both prey proteins, with 68 μM for Gab1 and 63 μM for SOS1. The ∼4-fold increase in peptide concentration compared to the reported value of 15 μM might be presumably caused by a lower affinity and blocking efficacy of SH3 domains in comparison to the SH2 domain.

Altogether, we could demonstrate that the assay is capable of determining putative differences in the specificity, efficacy, and affinity of known as well as unknown protein domain inhibitors.

### Monitoring Protein Complex Formation Dynamics in Individual Cells

It is now obvious that distinct PPI dynamics such as interaction lifetime, binding affinity, and protein complex stability are important regulators of fundamental processes in living cells. Therefore, the spatiotemporal manipulation and monitoring of signaling events is key to interlink the nature of dynamic signaling and its importance for information transfer and cell response.^58^ In order to learn how the cytosolic environment in a cell impacts protein complex formation and signaling rates, it is of particular importance to perform measurements in living cells rather than doing biochemical analysis in dilute solutions. Moreover, it is also appreciated to perform measurements on a single cell level to unravel cell-to-cell heterogeneities, as even genetically identical cells can behave differently.^18^

**Figure 7:**
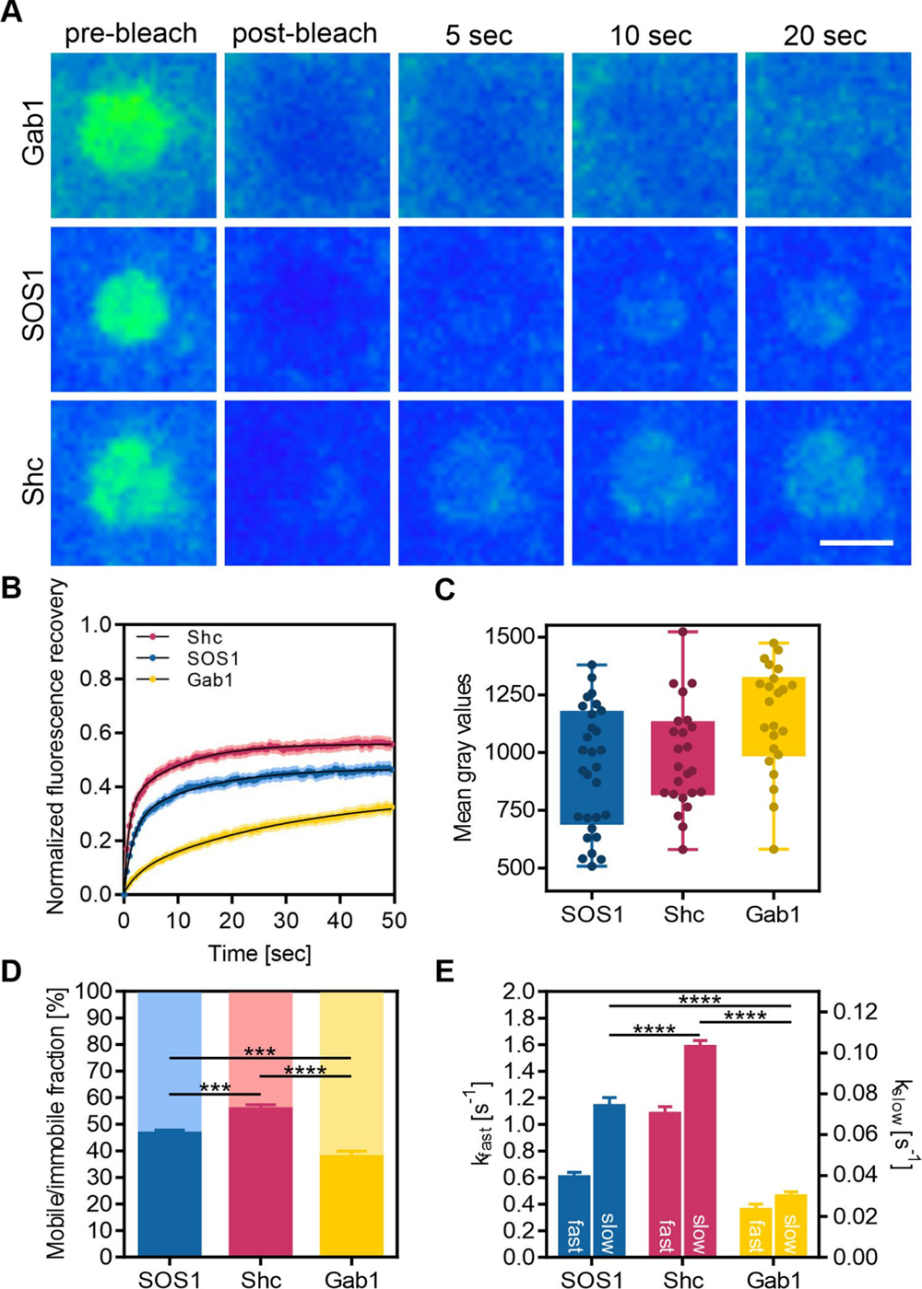
Monitoring protein complex formation dynamics in individual cells. (A) Representative TIR–FRAP images of single bleached prey patterns at indicated time points are shown. For FRAP experiments, cells were co-transfected with bait-PAR-Grb2 and indicated prey proteins and were grown on anti-HA patterned substrates. Prior to FRAP, cells were stimulated with EGF (170 nM) for at least 5 min. Individual patterns were selected for the FRAP experiment. Scale bar: 3 μm. Images shown were intensity adjusted and false colored for better visualization of differences in prey recovery dynamics. (B) Normalized mean fluorescence recovery curves of analyzed prey molecules. Black curves represent the two-component fit. (C) Mean fluorescence intensities of cells used for photobleaching experiments. (D) Calculation of mobile/immobile fraction. ***p < 0.001 and ****p < 0.0001 for comparison of mobile fractions. Dark bars represent mobile fraction, whereas light bars show the respective immobile fraction of the protein. (E) Calculation of exchange rates of prey molecules from fluorescence recovery curves. Diffusion of prey molecules is represented by *k*_fast_ (left *y*-axis). Prey binding to and dissociation from Grb2 is depicted as *k*_slow_ (right *y*-axis). ****p < 0.0001 for comparison of *k*_slow_. Error bars are based on mean ± SE of at least 25 analyzed cells measured on three different days.

As shown in previous studies, the micropatterning approach is a superior tool to study protein interaction kinetics in a live cell context.^10−12,28,59,60^ To monitor protein complex formation dynamics in individual cells, we carried out TIR-based fluorescence recovery after photobleaching (TIR–FRAP) experiments (Figure 7). We therefore used this approach to further elucidate the different observed interaction regimes. For this purpose, cells cotransfected with bait-PAR-Grb2 and different prey molecules were grown on anti-HA antibody patterned surfaces and single patterns were bleached using a high-intensity laser pulse for the determination of the temporal prey fluorescence recovery dynamics (Figure 7A). Figure 7B shows the respective fluorescence recovery curves for the indicated prey proteins. Depending on their lifetime, PPIs can be discriminated into permanent or transient interactions, whereas the latter ones are crucial for short-lived biological processes such as signal transduction.^61^ In general, the recovery process of the three investigated prey molecules (Shc1, SOS1, Gab1) proved to be fast, indicating transient PPIs. From the FRAP curves, the exchange rate of the freely diffusing pool of prey molecules into and out of the bleached ROIs was obtained through a bi-exponential fit as the slow recovery rate (*k*_slow_) (Figure 7E), whereas the fast recovery rate (*k*_fast_) represents free diffusion.^62^ A biphasic binding behavior of Grb2 to adapter proteins was previously reported.^63^ In living cells, such a two-step model could be described with an initial diffusion step of the adapter protein (here the prey protein) from cytosolic compartments to the membrane interface, followed by a second step including specific bait-prey binding/rebinding events. Interestingly, Gab1 and SOS1, which were found to be constitutively bound to Grb2, exhibited a significantly lower exchange rate (*k*_slow_) than Shc1, which was shown to interact with Grb2 in an agonist-dependent manner (Gab1: 0.030 ± 0.002 s^–1^, SOS1: 0.074 ± 0.004 s^–1^, and Shc1: 0.103 ± 0.003 s^–1^). Those results suggest half-times of dissociation from the pattern-bound immobile bait–prey associations of about 30 s for Gab1, 13 s for SOS1, and 10 s for Shc1. Moreover, the free diffusing pool of Gab1 and SOS1 was significantly lower than for Shc1. A possible explanation for the reduced fast diffusion of SOS1 and Gab1 in comparison to Shc1 might be that Gab1 and SOS1 are not existing in isolation, as they were reported to form stable SOS1:Grb2, Grb2:Gab1, and even SOS1:Grb2:Gab1 ternary complexes with different stoichiometries.^64,65^ It is likely, especially in the context of a cellular milieu, that the measured fast recovery kinetics were not obtained from isolated Gab1 and SOS1 molecules, but instead from stable macromolecular protein assemblies, which would diffuse slower than single Shc1 molecules in the cytosol. In the FRAP experiments, a portion of the prey molecules appeared immobile on a timescale of seconds as evidenced by the incomplete fluorescence recovery (Figure 7D). In line with the observation for different exchange rates, Gab1 and SOS1 showed significantly decreased mobile fractions (37.8 ± 2.1 and 46.7 ± 1.1%) when compared to Shc1 (55.8 ± 1.5%). The decreased exchange and mobile fraction of Gab1 and SOS1 molecules associated to the patterned Grb2 suggests either multiple association and dissociation events due to densely immobilized binding partners or indicates a more stable bait–prey association and protein complex stability. As the prey expression level might influence the recovery rates of the bleached molecules, cells with comparable prey expression were used for FRAP experiments (Figure 7C).

Whereas spatiotemporal modelling and characterization of binding events of cytosolic proteins to membrane receptors, and more precisely to the EGFR, are well described,^66^ respective kinetic information on cytosolic PPIs in living cells is missing. We are aware of the fact that both, bait and prey modification (e.g., both are fluorescence fusion proteins; and in the case of the bait a covalent tethering to the transmembrane fusion), and ectopic overexpression might distort the kinetics of interactions and/or compete with endogenous interactions. Nevertheless, we can here provide evidence that the temporal regulation of EGFR signaling networks is not solely regulated by unique recruitment and binding signatures of different scaffold proteins to the receptor. Much more it appears that these signaling processes are also defined through the interaction properties of the downstream molecules itself.

## Conclusions

In summary, our presented dynamic immunopatterning approach possesses the following advantageous features: (1) many cytosolic proteins of interest which are at least able to be located to or near the cell membrane interface may be copatterned by use of the bait-PAR construct system (analysis of proteins from intracellular locations other than the cytosol might require further adaption of the bait-PAR with respect to flexibility and range of the cytosolic domain); (2) the subcellular relocalization of signaling molecules in spatially defined micropatterns within single cells enables for in-depth investigation of intracellular protein complexes in its native environment with high specificity using TIRF or confocal microscopy; (3) a more structural characterization might be achieved when combining our subcellular micropatterning assay with high-resolution microscopy techniques such as single molecule microscopy^11,67^ or CryoEM;^68,69^ (4) experiments can be performed in living cells in real-time, using relatively simple and straightforward imaging techniques. Most other approaches addressing similar questions either require cell lysis, or even more sophisticated imaging modalities; (5) upon optimization of bait and prey fluorophore positions, resonance energy transfer-based experiments such as FRET, BRET, FLIM, and simultaneous FRAP and FRET are possible, which would enable a direct proof of PPIs; (6) the high-content platform based on microstructured COP foils enables for increased experimental throughput; (7) large-area μCP warrants a robust soft-lithography technique for modular protein patterning; (8) assay can be monitored with internal positive and negative controls to avoid false-positive results; and (9) assay can be easily implemented and adapted for different biological purposes.

Besides the presented methodological convenience, there are still some general experimental and biological considerations which must be taken into account: (1) μCP itself has methodological limitations compared to other surface patterning strategies; (2) multiplexing of different bait-PARs to simultaneously monitor multiple different interactions during receptor-induced signaling in the same cell is currently not realized; (3) modifications of bait and prey proteins (e.g., fusion to fluorescent proteins, covalent bait tethering to the transmembrane fusion, and so forth) are necessary; (4) overexpression of bait and prey fusion proteins might distort the kinetics of interactions and/or compete with endogenous interactions; and (5) to apply this assay, a general simplification of potentially much more complicated signaling interactions is necessary. We therefore recommend (especially for rather poorly described PPIs and signaling pathways) the combination of the immunopatterning approach with established biochemical and/or biophysical methods.

Nonetheless, we envision that the method described in this paper is a valuable alternative or add-on to standard wet laboratory-based technologies such as biochemical immunoprecipitation.

## Materials and Methods

### Reagents, Materials, and DNA Constructs

Bovine serum albumin (BSA), tyrphostin AG 1478, actinomycin D, indomethacin, streptavidin, EGF, (3-glycidyloxypropyl)trimethoxysilane (GPS) (98%), PDMS (SYLGARD 184), and DMSO were purchased from Sigma-Aldrich (Schnellendorf, Germany). BSA-Cy5 was obtained from Protein Mods (Madison, Wisconsin, USA). Biotinylated anti-EGFR, anti-HA, mouse-IgG, and anti-mouse-IgG (FITC) were purchased from Antibodies Online (Herford, Germany). The cell-permeable cyclic peptide cyclo(YpVNFΦrpPRR) was custom-synthesized by BioCat GmbH (Heidelberg, Germany) and the VPPPVPPRRR peptide was purchased from Santa Cruz Biotechnology (Dallas, Texas, USA). COP (Zeonor-COP) foils with a thickness of 100 μm were obtained from microfluidic ChipShop GmbH (Jena, Germany). 384-Well plastic castings were purchased from Greiner Bio-One GmbH (Frickenhausen, Germany). The following DNA constructs were kindly provided by the indicated persons: HA-RI-α-PARC-GFP (bait-PARC encoding regulatory subunit RI-α of protein kinase A) from Leif Dehmelt (MPI Dortmund, Germany), Gab1-GFP from Fred Schaper (Otto-von-Guericke-University Magdeburg, Germany), CFP-PI3K(p85α) from Shin-Ichiro Takahashi (University of Tokyo, Japan), Shc-RFP from John E Ladbury (University of Leeds, UK), GFP-SOS1 from Giorgio Scita (IFOM Milan, Italy), CFP-HRas from Philippe Bastiaens (MPI Dortmund, Germany), Raf1-CFP from Emilia Galperin (University of Kentucky, USA), MEK1-GFP from Rony Seger (Weizmann Institute of Science, Israel), sfGFP from Peter Pohl (JKU Linz, Austria) and Grb2-YFP from Lawrence E. Samelson (NIH Bethesda, USA).

### Construction of Bait-PAR-Grb2

To create arrays of cytosolic Grb2 (bait protein) inside living cells, a HA-PAR-Grb2 construct was generated that transfers the micrometer-scale antibody surface pattern. For this purpose, the regulatory subunit RI-α of the protein kinase A in the previously published HA-RI-α-PARC-GFP^20^ was replaced with Grb2 as the following: for seamless DNA insertion, the exponential megapriming PCR method was used for all cloning steps.^70^ The regulatory subunit RI-α was replaced with Grb2 by amplifying a 800 bp PCR product containing the Grb2 sequence flanked by sequences homologous to the 5′-site and the 3′-site of the HA-RI-α-PARC-GFP vector. The obtained product was purified (QIAquick PCR Purification Kit, Qiagen, Vienna, Austria) and used as a megaprimer in a second PCR run. In a second cloning attempt, the GFP tag was replaced by sfGFP using the identical strategy. Three linker sequences were inserted by round-the-horn PCR. The sequences of interest were divided in two halves and each site was used as a tag on a primer annealing at the respective site where the linker should be inserted. The blunt ends after PCR were ligated by T4 Ligase (Thermo Fisher, Linz, Austria). Linker sequences are depicted in Figure 3D.

### Cell Culture and Transfection

All cell culture reagents were purchased from Biochrom GmbH (Berlin, Germany). HeLa cells (ATCC) were cultured in a RPMI medium supplemented with 10% FBS and 1% penicillin/streptomycin and grown at 37 °C in a humidified incubator with 5% CO_2_. For transient transfection, cells were sub-cultured the day before and were then transfected with plasmids using the jetOPTIMUS DNA transfection reagent (Polyplus transfection, Illkirch, France), according to the manufacturer’s instructions.

### μCP

A PDMS stamp was replica molded by a casting PDMS prepolymer mixed in a ratio of 10:1 (component A/B) onto a photolithographically fabricated patterned silicon master. The silicon master (100 mm in diameter) containing a full array of round shaped pillars with a feature size and a depth of 3 μm was obtained from Delta Mask B.V. (Enschede, Netherlands). The PDMS stamp was peeled off the mask and stored at room temperature. The preparation of the micropatterned COP foil was carried out as the following: briefly, COP foils were washed with ethanol and dH_2_O before hydrophilization by plasma oxidation. Subsequently, hydrophilized COP foils were incubated overnight in GPS/ethanol (1:100, v/v) to form a monolayer of epoxide functional groups on the surface followed by washing with ethanol. For μCP, the large-area PDMS stamp was washed by flushing with ethanol (100%) and distilled water. After drying with nitrogen, the stamp was incubated in 50 mL BSA (or BSA-Cy5) solution (1 mg/mL) for 30 min. This step was followed by washing the stamp again with phosphate-buffered saline (PBS) and distilled water. After drying the stamp with nitrogen, the stamp was placed by its own weight on the clean epoxy-coated COP foil and incubated overnight at 4 °C. The next day, the stamp was carefully stripped from the substrate and the foil was bonded to a 384-well plastic casting using an adhesive tape (3M) and closed with an appropriate lid.

### Live Cell Micropatterning Experiments

For live cell experiments, selected 384-well reaction chambers were incubated with 20 μL/chamber streptavidin solution (50 μg/mL) for 30 min at room temperature. After washing two times with PBS, 20 μL/chamber biotinylated antibody solution (10 μg/mL) was added for 30 min at room temperature. Lastly, the incubation chambers were washed twice with PBS, and cells were seeded at defined cell density for the live cell microscopy analysis. The cells were allowed to attach to the surface for at least 3–4 h prior to imaging to ensure a homogeneous cell membrane/substrate interface, which is a prerequisite for quantitative TIRF microscopy.

### TIRF Microscopy

The detection system was set up on an epi-fluorescence microscope (Nikon Eclipse Ti2). A multilaser engine (Toptica Photonics, Munich, Germany) was used for selective fluorescence excitation of CFP, GFP, RFP, and Cy5 at 405, 488, 561, and 640 nm, respectively. The samples were illuminated in TIR configuration (Nikon Ti-LAPP) using a 60× oil immersion objective (NA = 1.49, APON 60XO TIRF). After appropriate filtering using standard filter sets, the fluorescence was imaged onto a sCMOS camera (Zyla 4.2, Andor, Northern Ireland). The samples were mounted on an *x*-*y*-stage (CMR-STG-MHIX2-motorized table, Märzhäuser, Germany), and scanning of the larger areas was supported by a laser-guided automated Perfect Focus System (Nikon PFS).

### TIR-FRAP Experiments and Calculation of Diffusion Coefficients

FRAP experiments were carried out on the epi-fluorescence microscope as described above. Single patterns were selected and photo-bleached (Andor FRAPPA) with a high-intensity laser pulse applied for 500 ms. Recovery images were recorded at indicated time intervals. Normalization of data was conducted by pre-bleach images, and first data analysis (quantitation of fluorescence recovery in single selected patterns) was carried out using NIS Elements software package (Nikon). Further data processing was performed in GraphPad Prism as described below. Resulting FRAP curves were plotted based on the standard error of the mean and fitted using a bi-exponential equation. Kinetic FRAP parameters were directly obtained from curve fitting using a diffusion-uncoupled two-component fit
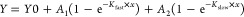
where *A*_1_ is the amplitude of the fast-diffusing population, *A*_2_ the amplitude of the slow diffusing population (binding reaction), and *k*_fast_ and *k*_slow_ are the rate constants of *A*_1_ and *A*_2_, respectively.

### Contrast Quantitation and Statistical Analysis

Contrast analysis was performed as described previously.^8^ In short, initial imaging recording was supported by the Nikon NIS Elements software. Images were exported as TIFF frames and fluorescence contrast analysis was performed using the Spotty framework.^71^ At first, 16-bit TIF images were imported in the micropatterning analysis software where an automatic gridding algorithm determines the grid parameters that correctly fit the micropatterned structure (mainly grid size and the rotation angle of the used image). The generated grid subdivides the total image into adjacent squares, each of which is quantified according to the average signal within a central circle comprising the micropattern spot (*F*^+^) and the signal outside this circle (*F*^–^). Based on the correct identification of the grid position, the fluorescence contrast ⟨*c*⟩ was calculated as ⟨*c*⟩ = (*F*^+^ – *F*^–^)/(*F*^+^ – *F*_bg_), where *F*^+^ denotes the intensity of the inner pixels of the pattern. *F*^–^ shows the intensity of the surrounding pixels of the micropattern and *F*_bg_ shows the intensity of the global background. Cells for quantitation were selected manually based on their morphology, size, and initial bait-patterning. In order to correct for putative differences in bait patterning, results were normalized for bait fluorescence contrast were indicated. For quantitation of bait–prey unbinding events and dose-response relationship (Figure 6E,F), bait-normalized data were further transformed (normalization between 0 and 100%) for better comparison between different treatment groups (inhibitors and prey proteins). For significance testing, an unpaired *t*-test was used to compare two experimental groups, whereas comparison of more than two different groups was performed using one-way ANOVA, which was followed by Tukey’s multiple comparisons test. All data transformation and statistical comparisons were carried out in GraphPad Prism software (version 7).

## Supporting Information Available

Impact of substrate patterning on bait and prey distribution and investigation of further signaling proteins of the Ras-Raf-MEK pathway (PDF)

## Notes

Associated preprint. The original manuscript was deposited on the preprint server bioRxiv: Hager Roland, Mueller Ulrike, Ollinger Nicole, Weghuber Julian, Lanzerstorfer Peter. Subcellular micropatterning for visual immunoprecipitation reveals differences in cytosolic protein complexes downstream the EGFR. 2021. bioRxiv. https://doi.org/10.1101/2021.05.25.445547.
