# Supplementary material for: Subcellular Dynamic Immunopatterning of Cytosolic Protein Complexes on Microstructured Polymer Substrates
Source: ACS Sens. 2021 Oct 15;6(11):4076–88. doi: 10.1021/acssensors.1c01574 (PMC8630788; doi:10.1021/acssensors.1c01574)
Supplement: Supplementary file 1 — se1c01574_si_001.pdf [file se1c01574_si_001.pdf]

Supporting Information

Subcellular dynamic immunopatterning of cytosolic protein complexes on microstructured polymer substrates

Roland Hager[1], Ulrike Müller[1], Nicole Ollinger[2], Julian Weghuber[1,2] and Peter Lanzerstorfer[1]**

[1]University of Applied Sciences Upper Austria, School of Engineering, 4600 Wels, Austria

[2]Austrian Competence Centre for Feed and Food Quality, Safety & Innovation, Head Office: FFoQSI GmbH, Technopark 1C, 3430 Tulln, Austria.

Correspondence to: peter.lanzerstorfer@fh-wels.at

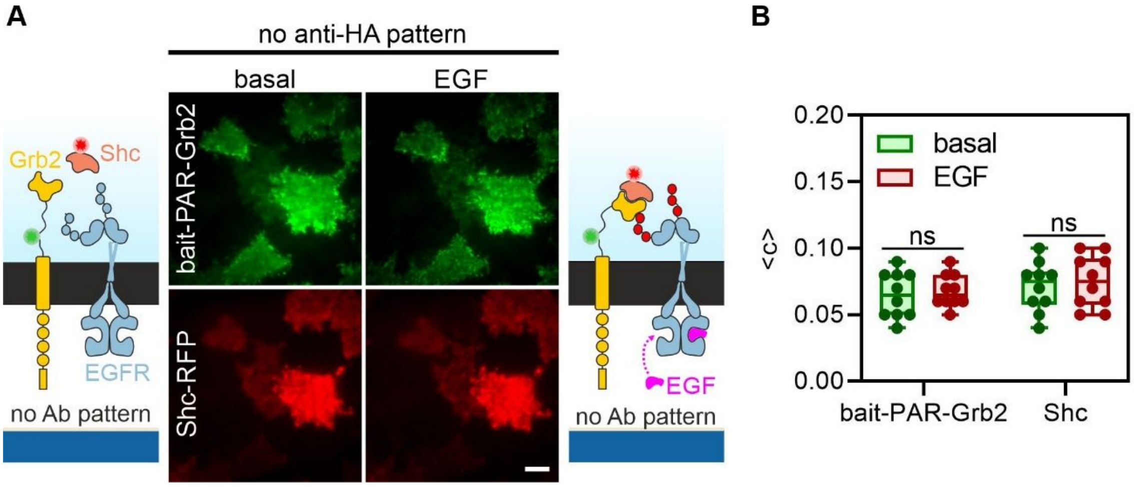

Figure S1. Impact of substrate patterning on bait and prey distribution. (A) Cells co-expressing bait-PAR-Grb2 (GFP-labelled) and Shc-RFP were grown on BSA-passivated COP substrates without antibody patterning. Scale bar: 15 µm. (B) Distribution of bait and prey was assessed by TIRF microscopy and quantitated before and after EGF stimulation (170 nM, 10 min). Error bars are based on the mean ± SE of 10 analyzed cells. ns, no significant difference between groups.

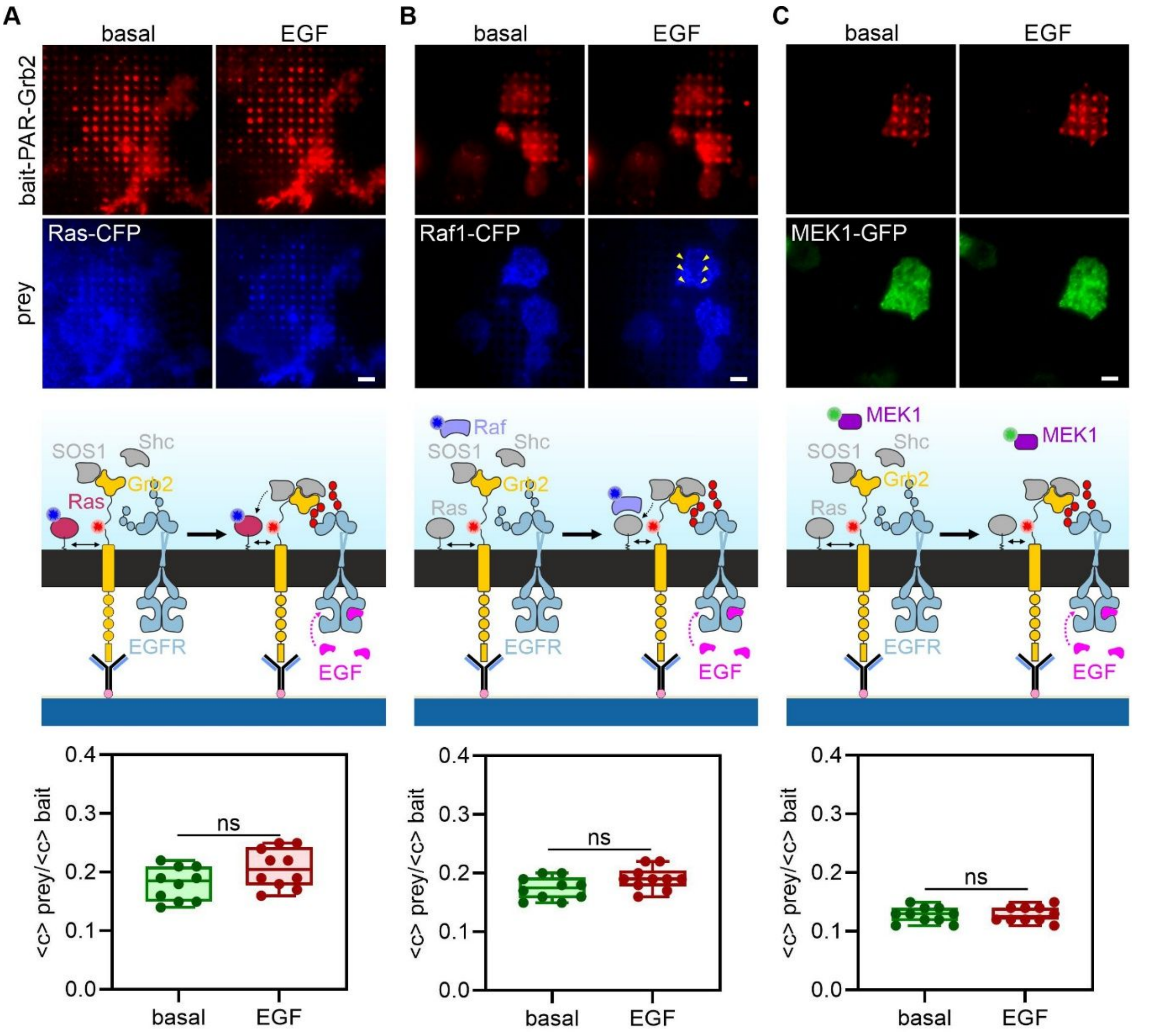

Figure S2. Investigating the Ras-Raf-MEK pathway. Representative TIRF microscopy images are shown of cells grown on anti-HA patterned substrates and co-expressing bait-PAR-Grb2 (RFP labelled) and (A) HRas-CFP, (B) Raf1-CFP, and (C) MEK1-GFP. Scale bar: 15 µm. Schematic presentations illustrate Ras-Raf-MEK downstream signalling pathway and formed protein complexes. Bar charts show quantitation of bait-normalized fluorescence contrast before and after EGF stimulation (170 nM, 10 min). Error bars are based on the mean ± SE of 10 analyzed cells. ns, no significant difference between groups.
